# O.2.2-6 ‘Let’s Ask the Service Users and Staff!’ - Barriers and Facilitators to Increasing Physical Activity in Medium Secure Psychiatric Services in the UK?

**DOI:** 10.1093/eurpub/ckad133.121

**Published:** 2023-09-11

**Authors:** Gloria Lui, Guy Faulkner, Simon Gibbon, Catherine Hewitt, Elizabeth Hughes, Kiara Lewis, Mike Lucock, Bal Singh, Phil Walters, Judith Watson, Tammi Walker

**Affiliations:** Durham University, UK; University of British Columbia; Nottinghamshire Healthcare NHS Foundation Trust; University of York; Edinburgh Napier University; Leeds Beckett University; University of Huddersfield; South West Yorkshire Partnership NHS Foundation Trust; gloria.m.lui@durham.ac.uk; South West Yorkshire Partnership NHS Foundation Trust; gloria.m.lui@durham.ac.uk; University of York; Durham University, UK

## Abstract

**Purpose:**

As part of a four-phase project, ‘**I**ncreasing Physical Activity in a **M**edium Service: The Development and Feasibility of a **P**hysical **ACT**ivity Intervention (IMPACT)’, the barriers and facilitators to increasing physical activity (PA) in medium secure psychiatric services, in the UK, were explored. Previous reviews have called for additional exploration into barriers and facilitators, as evidence has suggested a positive association between PA and the improvement in physical and psychological health for people with serious mental illnesses (SMI) and low activity levels.

**Methods:**

A mixed-method approach was adopted. Questionnaires, co-developed with service users, were completed across two NHS study sites in England, UK (n = 68), collecting qualitative and quantitative data from service users in medium secure services. Two focus groups were also conducted to collect qualitative data (n = 24) from hospital staff and key stakeholders. Framework Analysis and the COM-B Model of Behaviour Change (C for Capability, O for Opportunity, M for Motivation and B for Behaviour) were used to analyse the data. For this paper, the qualitative data from both the questionnaires and the focus groups will be discussed.

**Results:**

Similar themes were identified by the service users, hospital staff and key stakeholders. One main barrier discussed was environmental factors in secure services such as sedentary ward environments and the lack of autonomy in secure services. Main facilitators discussed were increasing the knowledge of benefits to PA and having structural guidance for personal development.

**Conclusion:**

The barriers and facilitators identified will inform the remaining phases of the IMPACT project, including a co-produced PA intervention, based on overcoming the barriers and supporting the facilitators identified. A feasibility study will then test the PA Intervention. This feature of co-producing a PA intervention in medium secure services, based on barriers and facilitators, adds to the limited literature in this area. The aim of the IMPACT project is to inform future a pilot study and policies.

**Support/Source:**

National Institute for Health and Care Research (NIHR) - Research for Patient Benefit Programme (IRAS ID 297420).

